# Impact of radiotherapy for nasopharyngeal carcinoma on carotid stenosis risk: a meta-analysis

**DOI:** 10.1016/j.bjorl.2022.03.001

**Published:** 2022-03-21

**Authors:** Huiqing Liang, Yuanyong Zhou, Wei Xiong, Suimin Zheng

**Affiliations:** Guangzhou University of Chinese Medicine, Shunde Hospital, Department of Imaging, Foshan, China

**Keywords:** Nasopharyngeal carcinoma, Radiotherapy, Carotid stenosis, Meta-analysis

## Abstract

**Objects:**

Radiotherapy (RT) serves as the most effective treatment for Nasopharyngeal Carcinoma (NPC) and can cause carotid stenosis. The aim of this study is to assess the impact of RT on carotid stenosis in NPC patients, as well as to explore the risk factors for significant carotid stenosis.

**Methods:**

Studies reporting the carotid stenosis in NPC patients who underwent RT were found on PubMed, Embase and Web of Science. Outcomes of our interest included incidence of overall/significant stenosis, Common Carotid Artery (CCA) stenosis, External Carotid Artery (ECA) stenosis, Internal Carotid Artery (ICA) stenosis, and risk factors for significant carotid stenosis.

**Results:**

Sixteen studies met the inclusion criteria and were included in this meta-analysis. Pooled estimate showed that RT was associated with a significantly higher incidence of overall stenosis (Risk Ratio [RR = 3.53], 95% CI: 2.32‒5.37; *p* <  0.001) and significant stenosis (RR = 7.06, 95% CI: 3.61‒13.79; *p* <  0.001) as compared with controls. Moreover, patients treated with RT had a significantly higher risk of stenosis in CCA (RR = 6.87, 95% CI: 4.08‒11.58; *p* <  0.001), ICA (RR = 3.43, 95% CI: 1.35‒8.73; *p* =  0.010), ECA (RR = 9.37, 95% CI: 2.06‒42.68; *p* =  0.004), and ECA/ICA (RR = 2.18, 95% CI: 1.52‒3.13; *p* < 0.001). Meta-analysis indicated that age (RR = 1.46, 95% CI: 1.05‒2.04; *p* =  0.024), smoking habit (RR = 1.20, 95% CI: 1.02‒2.78; *p* =  0.045) and time interval from radiotherapy (RR = 1.56, 95% CI: 1.07‒2.28; *p* =  0.02) were independent predictors of significant carotid stenosis.

**Conclusion:**

Our results suggested that RT increased the risk of carotid stenosis in patients with NPC. Prevention and control measurements should be made for older NPC patients with longer interval from RT, especially those with smoking habit.

**Level of evidence:**

3.

## Highlights

• Radiotherapy (RT) serves as the most effective treatment for Nasopharyngeal Carcinoma (NPC) and can cause carotid stenosis.
• This meta-analysis of 16 studies assessed the impact of RT on carotid stenosis in NPC patients, as well as to explore the risk factors for significant carotid stenosis.
• Results showed that RT increased the risk of carotid stenosis in patients with NPC. Age, smoking habit and time interval from radiotherapy were independent predictors of significant carotid stenosis.

## Introduction

Nasopharyngeal Carcinoma (NPC), a rare disease in the world, ranks the most common cause of head and neck cancer in southern China, where the incidence is 20‒40 per 100,000 person-years.^1^ Owning to the ongoing improvements in radiotherapeutic techniques and chemoradiotherapy, these patients achieved excellent local control and survival, even in those with locally advanced diseases.^2^ Because NPC is highly sensitive to radiation, Radiotherapy (RT) alone and concurrent chemo-radiotherapy are regarded as the most efficient treatment for early and advanced stages of disease.^3^ However, after irradiation, the late complications, such as optic neuropathy, brachial plexus injury and endocrine dysfunction are of great concern for patients and radiation oncologists.^4^

In patients with NPC, RT could damage the Carotid Artery (CA) and increase the risk of carotid atherosclerosis, which may result in transient ischemic attack and stroke.^5^ Compared patients with non-RT, post-RT NPC patients seemed to have a thicker CA wall, higher prevalence of carotid plaque and/or greater degree of carotid stenosis.^6^ It is reported that the post-RT patients have twice the risk of ischemic stroke compared with the non-irradiated patients. However, the arterial wall and plaque of carotid stenosis that induced by radiation are histologically similar to spontaneous atherosclerosis. Although previous studies have investigated the differences between irradiated patients and non-irradiated controls, the independent effects of RT on carotid atherosclerosis have been inconsistent among them, especially when the conventional cardiovascular risk factors were excluded. Therefore, we conducted this meta-analysis to investigate the effects of radiation therapy on carotid stenosis in NPC patients, as well as to explore the risk factors for significant carotid stenosis.

## Methods

### Literature search

We conducted this meta-analysis in accordance with the Preferred Reporting Items for Systematic Reviews and Meta-Analyses (PRISMA) guidelines.^7^ Four major electronic databases, including PubMed, Embase, and Web of Science, were comprehensively searched, from their inception to September 15, 2021. The literature search terms we used were the following: “Nasopharyngeal Neoplasm”, ‘‘Nasopharyngeal Carcinoma”, ‘‘Nasopharyngeal Cancer”, ‘‘Nasopharyngeal Tumor”, ‘‘carotid stenosis”, ‘‘plaque”, ‘‘atherosclerosis”, and ‘‘occlusion”. There were no limitations on publication status or language. We also additionally searched the reference lists of included articles and reviews to identify the potential eligible studies.

### Inclusion criteria and study selection

To be included in this meta-analysis, studies must meet the following inclusion criteria: (1) study design: Randomized Controlled Trials (RCTs), cohort study, case-control study or comparative study; (2) Population: adult patients who were histologically and/or clinically diagnosed NPC; (3) Intervention: RT; (4) Outcomes: number of patients with carotid stenosis, blood vessels affected, and the risk of factors associated with significant carotid stenosis.

### Data extraction and quality assessment

Using a standardized tool, two independent investigators extracted the following data from each study: first author’s name, year of publication, country, sample size, baseline patient characteristics, disease characteristics, radiotherapy dose, and the outcomes of our interest.

For non-randomized trials, we used the modified Newcastle-Ottawa Scale (NOS) to assess the methodological quality.^8^ This method comprised of three items to evaluate the quality of a non-RCT trial.^8^ The total score of this method was 9 points, and higher points indicated high quality. Studies with a score of more than 5 points were regarded as high quality.^8^

### Statistical analysis

Meta-analysis was analyzed using Stata version 12.0 software (Stata Corporation, College Station, TX, USA). We used Cochrane Q and I^2^ statistic^9^ to test the heterogeneity across included studies, in which *p* <  0.1 or I^2^ > 50% were considered to be significant.^9^ For dichotomous variables, Risk Ratio (RR) with 95% Confidence Intervals (CIs) was pooled to synthesize the data. Meta-analyses were performed using a fixed-effect model^10^ or random-effects model^11^ according to the absent or present of heterogeneity. When significant heterogeneity analysis was identified, we used sensitivity analysis to explore the potential sources of heterogeneity. Moreover, we also conducted subgroup analysis based on blood vessel and area affected to test whether it had an influence on the outcome estimate. We also performed meta-analysis by pooling data from logistic regression analysis of risk factors to identify whether they were associated with significant carotid stenosis. The assessment of publication bias was evaluated by using Egger^12^ and Begger^13^ test. A *p*-value less than 0.05 was judged as statistically significant, except where otherwise specified.

## Results

### Identification of eligible studies

**Figure 1:**
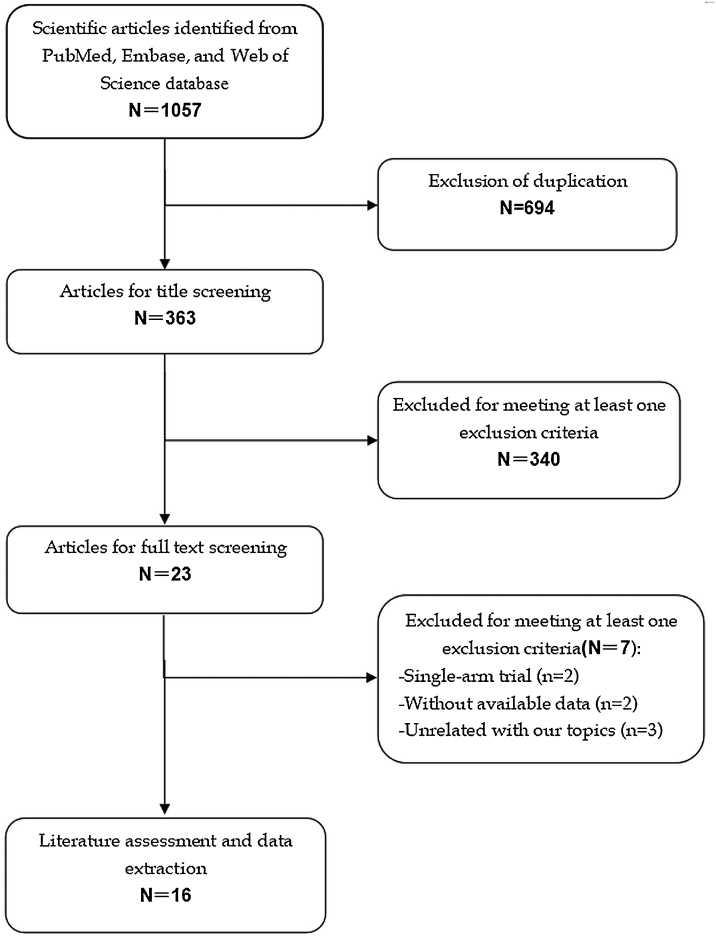
Eligibility of studies for inclusion in meta-analysis.

The initial screening retrieved 1057 publications from the databases, of which 694 were excluded because of duplicate records, leaving 363 studies. Further screening for title or abstract excluded 340 studies, leaving 23 for full-text information review. Among these studies for potential eligibility, 7 studies were excluded for a variety of reasons. Finally, 16 studies^14^, ^15^, ^16^, ^17^, ^18^, ^19^, ^20^, ^21^, ^22^, ^23^, ^24^, ^25^, ^26^, ^27^, ^28^, ^29^ met the inclusion criteria and were included for the data analysis. The literature review and selection process are presented in Fig. 1.

### Characteristics of eligible studies and quality assessment

Table 1 summarizes the characteristics of the included studies. Among these studies, 14 were performed in China,^14^, ^15^, ^16^, ^17^, ^18^, ^19^, ^20^, ^21^, ^23^, ^24^, ^25^, ^26^, ^28^, ^29^ and the remaining two studies were in Italy^22^ and Malaysia,^27^ respectively. These studies were published between 1998 and 2018, 8 of which were case-control studies,^21^, ^23^, ^24^, ^25^, ^26^, ^27^, ^28^, ^29^ 6 were cross-sectional studies^15^, ^16^, ^17^, ^18^, ^19^, ^20^ and 2 were cohort studies.^14^, ^22^ The radiotherapy dose varied greatly, ranging from 4500 cGy to 8100 cGy. Of the included studies, 10 used NPC without RT as control and 6 used healthy subjects as control. Sample size ranged from 90 to 319, with a total of 2646. The mean age of patients in the included studies was 53.7 ± 5.16 years old, and 66.83% of the enrolled patients were male. The interval from RT in most patients was more than 0.3 years, with a maximum duration of 30 years.

**Table 1:** Baseline characteristics of patients in the trials included in the meta-analysis.

Study	Country	Year of publication	Study design	Radiotherapy dose (Gy)	Subjects	Nº of patients	Interval from RT (years)	Male/Female	Age (mean ± SD, y)	NOS score
Zhou L^14^	China	2015	Cohort	66.0	NPC	72	5.7 (3‒16)	44/28	54 (19‒81)	7
					NPCWR	50		29/21	54 (20‒85)	
Chang YJ ^15^	China	2019	Cross-sectional study	6000cGy	NPC	192	2.0 (0.3‒19.1)	139/53	49.9 ± 11.7	6
					NPCWR	98		71/27	49.8 ± 12.5	
Cheng WS^16^	China	1999	Cross-sectional study	5500cGy	NPC	85	6.4 ± 5.5	NR	59.3 ± 14.0	5
					NPCWR	108		NR	62.1 ± 10.3	
Cheng WS^17^	China	2000	Cross-sectional study	60‒66	NPC	96	6.7	NR	53.6	5
					NPCWR	96		NR	61.8 ± 10.5	
Dubec JJ^18^	Canada	1998	Cross-sectional study	5950cGy	NPC	45	NR	30/15	67	6
					HS	45		NR	NR	
Huang TL^19^	China	2013	Cross-sectional study	68.4‒78.6	NPC	105	4.0	72/33	52.43 ± 10.23	5
					HS	25		16/9	50.68 ± 11.49	
Lam WW^20^	China	2001	Cross-sectional study	56.4	NPC	71	4‒20	53/18	53.6 (38‒64)	6
					NPCWR	51		35/16	48.8 (26‒87)	
Lam WW^21^	China	2002	Case-control study	56.6	NPC	71	4‒11	52/19	53.6 (39‒69)	7
					NPCWR	142		91/51	60.6 (20‒83)	
Greco A^22^	Italy	2012	Cohort study	50‒60	NPC	39	NR	31/8	62.1	5
					NPCWR	54		40/14	63.7	
Li CS^23^	China	2010	Case-control study	4500‒8100 cGy	NPC	43	5	31/12	56 ± 7	5
					HS	276		166/110	64 ± 12	
Liao W^24^	China	2018	Case-control study	66.5 ± 4.7	NPC	96	6.3	73/23	51 (29‒75)	6
					NPCWR	137		113/24	52 (24‒72)	
Ye JH^25^	China	2012	Case-control study	60‒64	NPC	91	4.53 ± 1.69	66/25	38.29 ± 4.66	7
					HS	29		21/8	38.10 ± 5.35	
Chu PY^26^	China	2015	Case-control study	NR	NPC	19	14.8 (2‒30)	NR	64.9 ± 11.6	5
					NPCWR	133		NR	64.9 ± 11.6	
Tai SML^27^	Malaysia	2013	Case-control study	66	NPC	47	6.4 ± 7.9	31/16	55.1 ± 12.4	6
					HS	47		31/16	55.0 ± 12.9	
Yuan C^28^	China	2017	Case control study	66.87 ± 3.45	NPC	69	≥4	28/41	52.6 ± 8.4	7
					HS	76		37/39	42.8 ± 15.5	
Lam WW^29^	China	2001	Case control study	56.6	NPC	80	4‒26	NR	53 (38‒69)	6
					NPCWR	58		NR	53 (38‒69)	

SD, Standard Deviation; NR, Not Reported; RT, Radiotherapy; NPC, Nasopharyngeal Carcinoma; HS, Healthy Subjects; NPCWR, Nasopharyngeal Carcinoma Patients Without Radiotherapy.

The methodological assessment for cohort studies showed that, the NOS score in each study was greater than 5 points, indicating that they were of high quality.

### Carotid stenosis

**Figure 2:**
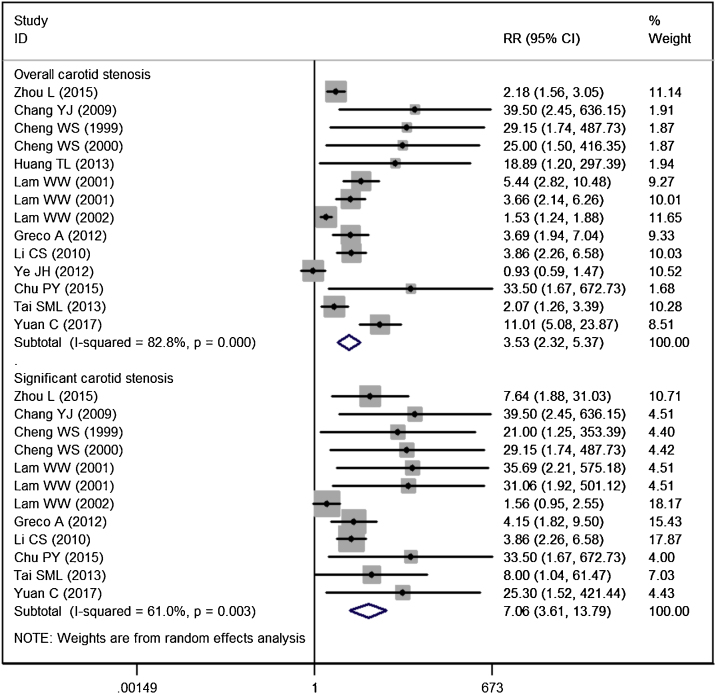
Forest plot showing the effect of RT on the overall/significant stenosis in patients with NPC.

Fourteen studies reported the data of carotid stenosis.^14^, ^15^, ^16^, ^17^, ^19^, ^20^, ^21^, ^22^, ^23^, ^25^, ^26^, ^27^, ^28^, ^29^ The incidence of overall stenosis in RT group and control group was 47.59% and 18.26%, respectively. Pooled estimate suggested that NPC patients treated with RT had a significantly higher incidence of overall stenosis than the control (RR = 3.53, 95% CI: 2.32‒5.37; *p* <  0.001) (Fig. 2). The test for heterogeneity showed a significant difference among the included studies (I^2^ = 82.8%, *p* <  0.001). Therefore, we performed sensitive analysis by excluding the trial with outlier,^25^ and results showed the overall estimate (RR = 4.06, 95% CI: 2.65‒6.23; *p* <  0.001) and heterogeneity (I^2^ = 80.5%, *p* <  0.001) did not change substantially. We then excluded the trial with small sample size,^26^ and the overall estimate did not change substantially (RR = 3.38, 95% CI: 2.23‒5.13; *p* <  0.001), but the heterogeneity was still present (I^2^ = 83.4%, *p* < 0.001).

The incidence of significant stenosis in RT group and control group was 22.82% and 5.55%, respectively. The incidence of significant stenosis in RT group was significantly higher than that in control group (RR = 7.06, 95% CI: 3.61‒13.79; *p* <  0.001). There was significant heterogeneity across the included studies (I^2^ = 61.0%, *p* =  0.003). When we then excluded the trial with outlier,^21^ the overall estimate did not change substantially (RR = 7.25, 95% CI: 4.23‒12.42; *p* <  0.001), but the heterogeneity was not present (I^2^ = 19.1%, *p* = 0.261). This indicated that the trial conducted by Lam WW, et al.^21^ was responsible for the heterogeneity.

### Subgroup analysis by blood vessel and area

#### CCA stenosis

**Figure 3:**
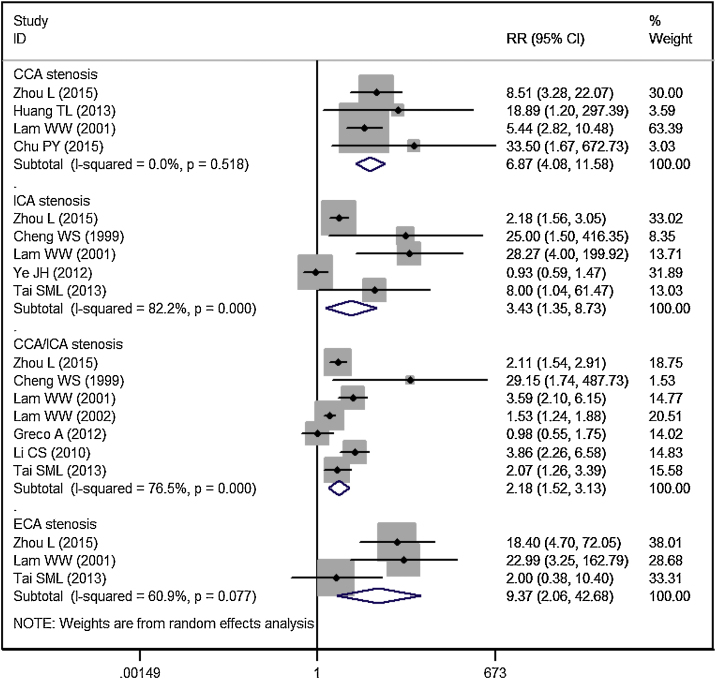
Forest plot showing the effect of RT on the stenosis in CCA, ICA, ECA, and CCA/ICA in patients with NPC.

Eleven studies reported the data of carotid stenosis by blood vessel and area affected.^14^, ^15^, ^16^, ^19^, ^20^, ^21^, ^22^, ^23^, ^25^, ^26^, ^27^ The incidence of overall CCA stenosis in RT group and control group was 53.99% and 4.51%, respectively. Patients treated with RT had a significantly higher risk of CCA stenosis than controls (RR = 6.87, 95% CI: 4.08‒11.58; *p* <  0.001) (Fig. 3).

**Figure 4:**
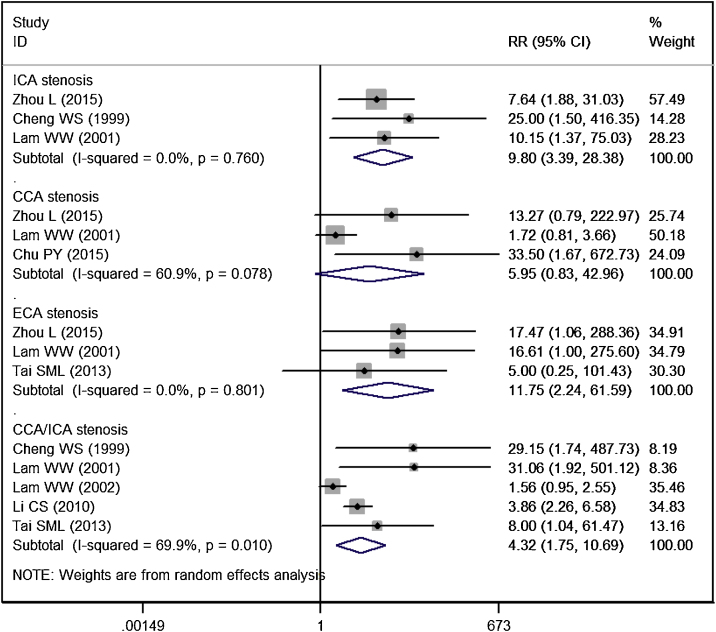
Forest plot showing the effect of RT on the significant stenosis in CCA, ICA, ECA, and CCA/ICA in patients with NPC.

The incidence of significant CCA stenosis in RT group and control group was 17.54% and 3.32%, respectively. Patients treated with RT had a similar risk of significant CCA stenosis than controls (RR = 5.95, 95% CI: 0.83‒42.96; *p* =  0.077) (Fig. 4).

#### ICA stenosis

The incidence of overall ICA stenosis in RT group and control group was 42.99% and 25.52%, respectively. Patients treated with RT had a significantly higher risk of ICA stenosis than controls (RR = 3.43, 95% CI: 1.35‒8.73; *p* =  0.010) (Fig. 3).

The incidence of significant ICA stenosis in RT group and control group was 19.35% and 1.47%, respectively. Patients treated with RT had a significantly higher risk of significant ICA stenosis than controls (RR = 9.80, 95% CI: 3.39‒28.38; *p* < 0.001) (Fig. 4).

#### ECA stenosis

The incidence of overall ECA stenosis in RT group and control group was 46.84% and 3.38%, respectively. Patients treated with RT had a significantly higher risk of ECA stenosis than controls (RR = 9.37, 95% CI: 2.06‒42.68; *p* =  0.004) (Fig. 3).

The incidence of significant ECA stenosis in RT group and control group was 13.16% and 0.0%, respectively. Patients treated with RT had a significantly higher risk of significant ECA stenosis than controls (RR = 11.75, 95% CI: 2.24‒61.59; *p* =  0.004) (Fig. 4).

#### CCA and ICA stenosis

The incidence of overall CCA/ICA stenosis in RT group and control group was 58.53% and 22.73%, respectively. Patients treated with RT had a significantly higher risk of CCA/ICA stenosis than controls (RR = 2.18, 95% CI: 1.52‒3.13; *p* < 0.001) (Fig. 3).

The incidence of significant CCA/ICA stenosis in RT group and control group was 24.26% and 9.29%, respectively. Patients treated with RT had a significantly higher risk of significant CCA/ICA stenosis than controls (RR = 4.32, 95% CI: 1.75‒10.69; *p* =  0.002) (Fig. 4).

#### Vessel stenosis

**Figure 5:**
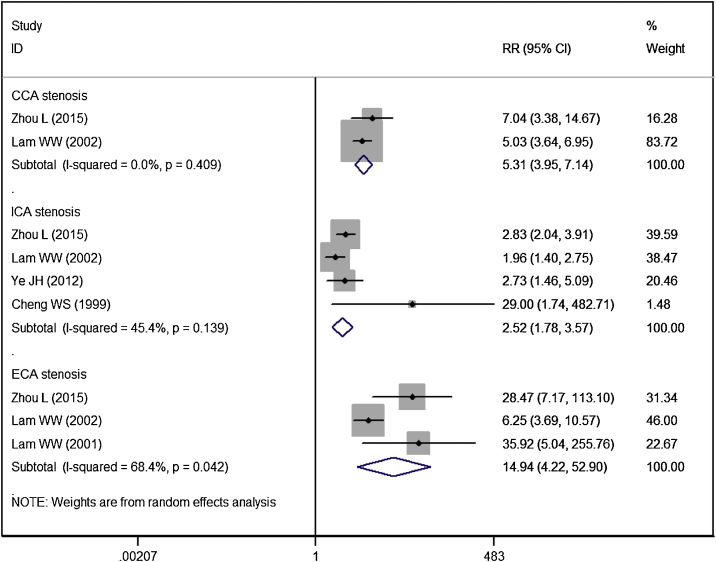
Forest plot showing the effect of RT on the carotid stenosis (vessel) in CCA, ICA and ECA in patients with NPC.

Five studies reported the data of vessel stenosis.^14^, ^16^, ^20^, ^21^^,^^25^ Patients in RT group had a significantly higher risk of CCA stenosis (RR = 5.31, 95% CI: 3.95‒7.14; *p* <  0.01), ICA stenosis (RR = 2.52, 95% CI: 1.78‒3.57; *p* <  0.01), ECA stenosis (RR = 14.94, 95% CI: 4.22‒52.90; *p* <  0.01), as compared to controls (Fig. 5).

### Risk factors for significant carotid stenosis

Nine of the included studies performed logistic regression analysis to identify risk factors associated with significant carotid stenosis.^14^, ^15^, ^17^, ^18^, ^19^, ^20^, ^23^, ^24^, ^26^ Then we performed meta-analysis to explore whether these variables could predict the significant carotid stenosis. Pooled data showed that, age (RR = 1.46, 95% CI: 1.05‒2.04; *p* =  0.024), smoking habit (RR = 1.20, 95% CI: 1.02‒2.78; *p* = 0.045) and time interval from radiotherapy (RR = 1.56, 95% CI: 1.07‒2.28; *p* =  0.02) were found to be independent predictors of significant carotid stenosis, and other variables did not have significant influence on this outcome (Table 2).

**Table 2:** Risk factors for significant carotid stenosis.

Variable	RR	95% CI	*p*
Age	1.46	1.05‒2.04	0.024
Smoking habit	1.20	1.02‒2.78	0.045
Time interval from RT	1.56	1.07‒2.28	0.020
Gender	1.32	0.72‒2.41	0.365
RT dose	0.02	0.01‒13.61	0.249
Hypertension	1.80	0.61‒5.29	0.289
Diabetes mellitus	1.50	0.68‒3.31	0.317
Heart disease	6.45	0.25‒166.28	0.261
Hyperlipdemia	1.77	0.70‒4.43	0.226
Surgery	0.27	0.03‒2.20	0.222

RR, Risk Ratio; 95% CI, 95% Confidence Interval; RT, Radiotherapy.

### Publication bias

Publication bias was assessed, and results showed that no publication bias was found (Egger’s test, *p* =  0.315; Begg’s test, *p* =  0.472).

## Discussion

In the present study, we performed a meta-analysis to comprehensively assess the effects of radiation therapy on carotid stenosis in NPC patients, as well as to explore the risk factors for significant carotid stenosis. Our results showed that NPC patients treated with RT had a significantly higher risk of overall/significant stenosis than the controls. Moreover, in the subgroup analysis based on the blood vessel and area affected, RT was associated with significantly higher incidences of stenosis in CCA, ICA, ECA and CCA/ICA than control. Age, smoking habit and time interval from RT were independent predictors of significant carotid stenosis.

CA stenosis is a common complication of external irradiation in head and neck cancers.^20^ Previous studies reported that the incidence of CA stenosis was increased more than 5-years after RT,^16^, ^30^ but some other studies found that significant CA stenosis could occur as early as 1‒2-years after RT.^31^, ^32^ Therefore, studies with different duration of follow-up might lead to different results. In this study, we included 16 studies of NPC patients, in which the effect of RT on carotid stenosis was assessed at different periods. Even though, our pooled data suggested that RT increased the risk of overall/significant stenosis. The exact mechanism of RT-induced CA disease is not clear, however, three possible reasons might account for this: (1) Fibrosis due to a damaged vaso vasorum, (2) Adventitial fibrosis producing obstruction, (3) Accelerated atherosclerosis.^33^ Cheng SW, et al.^34^ performed a prospective study and reported that, the carotid stenosis associated with external irradiation progressed more rapidly than nonirradiated atherosclerotic arteries.^34^ In that study, the annualized progression rate in irradiated arteries was higher than that in nonirradiated arteries (15.4% vs. 4.8%).^16^ The authors concluded that the carotid stenosis induced by external irradiation is not only attributed to pre-mature atherosclerosis alone, but also a more aggressive disease with a different biological behavior.^34^

In this study, we found that the incidence of overall/significant stenosis in NPC patients who underwent RT was higher than that of the controls. Our results were in accordance with the findings of previous studies.^14^, ^15^, ^20^ Lam WW, et al.^20^ examined the incidence of stenosis in the extracrial carotid arteries among NPC patients after RT. They found that the CCA/ICA was more common seen in RT group than in the non-RT group (77.5% vs. 21.6%, *p* <  0.01), followed by ECA (45.1% vs. 2.0%, *p* <  0.01) and Vertebral Artery (VA) (7.0% vs. 0%, *p* =  0.069).^20^ In addition, significant stenosis was only found in the RT group (CCA/ICA: 29.58%, ECA: 15.49%, VA: 5.63%).^20^ Therefore, the authors suggested that RT increased the risk of significant carotid stenosis.

Although a variety of studies have confirmed the relationship between RT and carotid stenosis, the incidence of significant stenosis induced by RT varies greatly among them. Carmody BJ, et al.^31^ and Lam WW, et al.^20^ reported that the rate of significant stenosis (70%‒90% and ≥ 50% stenosis, respectively) in RT group was 21.7% and 77.5%, respectively, which was higher than that in control groups (4% and 14.3%). The mean radiation dose in these studies was approximately 6000 cGy. Whereas, Chang YJ, et al.^15^ reported the rate of significant stenosis induced by RT (6225 cGy) was 19.8%, which was lower than the findings of the prior two studies. The different results in the three studies might be caused by the time interval between RT and examination, which was 6.5 years, 9.2 years, and 4.9 years in the studies of Carmody BJ, Lam WW and Chang YJ, respectively.

Another reason for the difference in incidence of carotid stenosis might be the different measurement methods. Zhou L et al.,^14^ reported that the incidence of CA and VA stenosis in RT group was 73.6% and 81.9%, which was higher than that of 45%/38% and 51.25%/7.04% in the two studies conducted by Law WW, et al.^20^, ^29^ In the two latter studies, ultrasonography was used to detect the degree of vessel stenosis by measuring the percentage reduction in the diameter of true lumen.^20^, ^29^ Whereas, in the prior study, the authors used Contrast-Enhanced MR Angiography (CE-MRA) to assess the degree of vessel stenosis by measuring the percentage reduction in the area of true lumen.^14^ It is reported that area measurement is closely related to the results of Digital Subtraction Angiography (DSA), which is more accurate than the diameter assessment, especially for arteries with irregular lumens.^35^ Moreover, ultrasonography cannot provide 3D image or detect intracranial CA stenosis. However, MIP images of CE-MRA can provide multiple projections of the CAs and can display a panoramic review of the Cas.^36^

In this study, we also investigated the risk factors for significant stenosis in patients with NPC after RT. Our results demonstrated that age, smoking habit and time interval from RT were independent predictors of significant carotid stenosis. These findings were in consistent with the results of previous studies.^24^ Liao W, et al.^24^ found that age was correlated with significant carotid stenosis in patients undergoing RT, and the age in RT group (54.1 ± 9.5 years) was significantly older than those in non-RT group (50.7 ± 8.0 years) (*p* =  0.015). Similarly, Cheng SW, et al.^17^ revealed that patients who had undergone RT more than 5-years earlier had a 15 times higher risk of developing significant carotid stenosis than those with less than 5-years (26% vs. 6%, *p* <  0.01). Chu PY, et al.^26^ found that smoking increased the incidence of extracranial artery stenosis in patients with NPC who underwent RT (adjusted Odd Ratio [OR = 4.472], 95% CI: 2.057‒9.725; *p* <  0.001). For the time interval from RT, both the studies conducted by Liao W, et al.^24^ and Zhou L, et al.^14^ showed that, it was associated with higher carotid stenosis risk (OR = 1.068, 95% CI: 1.033‒1.105, *p* =  0.001; β = 1.076, 95% CI: 0.998‒8.621, *p* =  0.05). Other risk factors, such as RT dose, hypertension, diabetes mellitus, heart disease, hyperlipidemia, and surgery, were not found to be related with the incidence of significant stenosis in this study. These findings were also in accordance with the results of other studies.^23^, ^26^ Even so, management of these risk factors is still beneficial for the patients with NPC who underwent RT since it is applicable to the general population.^37^

There were several potential limitations to note when interpreting our findings. First, the studies included in this meta-analysis were performed with observational design (cohort study, case-control study, or cross-sectional study). Although observational study is more likely to reflect the real-world, it is limited by the selection bias and potential for confounding factors. Second, there were moderate or substantial heterogeneity observed among the included studies. However, the source of heterogeneity for overall carotid stenosis was not identified after sensitivity analysis. This might be explained by the differences across the included studies, such as study design, selection of control, imaging techniques used for carotid stenosis, RT methods, and radiation dose. These factors may account for the heterogeneity and have an impact on the effects. Third, the sample size varied greatly among the included studies, some of which were relatively small. It is reported that studies with small sample size were more likely to overestimate the treatment effect as compared with larger trials. Therefore, large-scale trials are needed to draw definitive conclusions.

## Conclusion

The present meta-analysis suggested that patients who underwent RT of NPC had a higher risk of developing carotid stenosis. Thus, patients and physicians should be aware of the complications after RT so that every effort can be made to avoid the development of these complications. Considering the potential limitations in this study, more large-scale, well-designed trials are needed to verify our findings as well as to explore the mechanism for radiation-induced carotid stenosis.

## Conflicts of interest

The authors declare no conflicts of interest.
